# Bottlebrush anthocyanin-mediated green synthesis of ZnO nanoparticles and their integration into functional paper for multifunctional ammonia sensing and food quality monitoring

**DOI:** 10.1039/d6ra00160b

**Published:** 2026-05-19

**Authors:** Arati Dubey, Juhi, Poonam Sharma, Avanish Shukla, Adhish Jaiswal, Lebogang Katata-Seru, Indra Bahadur

**Affiliations:** a Department of Chemistry, Indira Gandhi National Tribal University Amarkantak Madhya Pradesh 484887 India jaiswal_adhish@lkouniv.ac.in avanish.shukla@igntu.ac.in bahadur.indra@nwu.ac.za; b Department of Zoology, Indira Gandhi National Tribal University Amarkantak Madhya Pradesh 484887 India; c Department of Chemistry, University of Lucknow Lucknow Uttar Pradesh India; d Department of Chemistry, North-West University (Mafikeng Campus) Private Bag X2046 Mmabatho 2735 South Africa

## Abstract

Green synthesis has garnered significant attention in recent years due to its sustainable and environment friendly approach to nanoparticle production. Numerous studies have explored the use of plant extracts as both reducing and stabilizing agents in the synthesis of zinc oxide (ZnO) nanoparticles (NPs). Here, Bottlebrush flower extract acts as a capping agent and reducing agent, as evidenced by its presence on the surface of ZnO nanoparticles. Absorption spectroscopy and X-ray Diffraction (XRD) techniques were used to characterize the bio-synthesized ZnO nanoparticles. The mean zeta potential value indicates that the prepared nanoparticles are moderately stable. The hydrodynamic diameter of zinc oxide nanoparticles in suspension was measured by DLS techniques. TEM analysis shows that the average particle size of the synthesized nanoparticles is 32 nm. The antioxidant activity of ZnO nanoparticles is also observed, which increases with increase in concentration of ZnO NPs. The synthesized ZnO NPs show antibacterial and antioxidant activity also. The fabricated ZnO NPs demonstrate high sensitivity and selectivity towards ammonia, making them highly suitable for food applications. A ZnO based paper sensor was successfully employed to check chicken freshness through color response test. Thus, bio derived ZnO nanomaterials serve as an environmentally friendly, cost effective, sustainable solution in sensing applications.

## Abstract 2

Green synthesis has garnered significant attention in recent years due to its sustainable and environment friendly approach to nanoparticle production.

## Graphical abstract

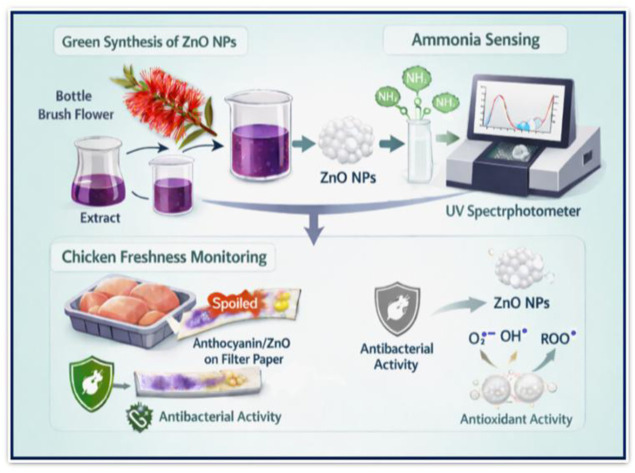

## 1. Introduction

The provision of safe and adequate nutrition is an essential human necessity and a fundamental component of public health. Despite significant advancements in food safety technologies and regulatory frameworks, foodborne diseases remain a major global health concern.^1–4^ Since early civilization, fermentation has been widely utilized to improve the shelf life, safety, and sensory properties of foods through the activity of selected microorganisms and their metabolic products. However, certain microorganisms present in food matrices can promote spoilage and pose serious health risks. Microorganisms that cause disease in humans after ingestion through contaminated food are known as foodborne pathogens.^5–7^ Foodborne diseases result from the consumption of food or water contaminated with pathogenic microorganisms, including bacteria, viruses, fungi, and certain parasitic organisms.^8,9^ Among these, bacterial pathogens such as *Escherichia coli*, *Salmonella enterica*, *Staphylococcus aureus*, *Listeria monocytogenes*, *Campylobacter jejuni*, *Bacillus cereus*, Shiga toxin-producing *Escherichia coli*, and species of the genus *Vibrio* are frequently associated with foodborne outbreaks. While infections caused by these pathogens are often mild and self-limiting in healthy individuals, vulnerable populations may experience severe complications, including systemic infections, pregnancy-related complications, and in extreme cases, death.^8,9^

To ensure food safety, various analytical methods and commercial detection kits have been developed for identifying foodborne pathogens. In routine quality control practices, the food industry regularly monitors microbial contamination at different stages of food processing and distribution.^10,11^ Conventional culture-based techniques remain the standard method for pathogen identification. These approaches involve morphological observation, biochemical characterization, and immunological analysis, usually performed before and after an enrichment step.^12^ In this process, the sample is introduced into a nutrient medium, incubated to allow microbial growth, and subsequently plated for isolation and examination.^13^ Although these methods are reliable, they are often time-consuming, labor-intensive, and costly, and they require specialized laboratory facilities.

In contrast, biosensor-based detection strategies have attracted considerable attention due to their rapid response, high sensitivity, and operational simplicity.^14^ Biosensors can be classified based on their signal transduction mechanisms, including electrochemical, gas-sensing, optical, magnetic, and thermoelectric systems. These devices convert biological or chemical interactions into measurable signals, such as changes in electrical resistance, capacitance, refractive index, or current.^15,16^ Among these techniques, gas-sensing technology has emerged as a promising approach for the rapid detection of food spoilage indicators under ambient conditions.^17^ Gas sensors operate by monitoring variations in electrical resistance when the sensing material interacts with specific gaseous analytes.^18^ These platforms offer several advantages, including high sensitivity, low detection limits, good selectivity, strong reproducibility, and long-term stability, making them suitable for real-time monitoring of food quality.^19^

Gas-sensing materials are generally categorized into five major groups: metal oxides, carbon-based nanomaterials, conductive polymers, transition metal chalcogenides, and metal-based hybrid nanostructures.^20^ Among these materials, metal oxides have attracted significant interest due to their low cost, simple fabrication processes, excellent surface reactivity, and high sensing performance.^21–23^ Metal oxide sensors exhibit rapid response times, strong selectivity, and the ability to detect a wide range of gases, including volatile organic compounds (alcohols, aldehydes, and ketones), hydrogen, nitrogen dioxide, ammonia, carbon dioxide, and hydrogen sulfide.^24–26^

In food safety applications, the detection of spoilage-related gases such as ammonia is particularly important. Ammonia is widely recognized as a key indicator of food spoilage, especially in protein-rich foods such as meat, fish, and dairy products.^27–30^ During storage, microbial metabolism and enzymatic protein degradation generate volatile basic nitrogen compounds, including ammonia, trimethylamine, and dimethylamine.^31–33^ Among these compounds, ammonia is one of the primary by-products associated with protein decomposition and the deterioration of food quality. The accumulation of ammonia not only produces unpleasant odors but also indicates microbial spoilage that can compromise food safety and consumer acceptance. Therefore, rapid and reliable ammonia detection is essential for monitoring food freshness and preventing the consumption of spoiled products.^34–36^

Metal oxide semiconductor (MOS) gas sensors have been extensively investigated for gas detection applications due to their high sensitivity and fast response characteristics. These sensors are also valued for their operational stability and versatile fabrication possibilities, including single crystals, thin films, thick films, nanorods, nanofilms, and nanoparticle-based structures.^37^ Among various metal oxide materials, zinc oxide (ZnO) has emerged as a promising candidate for gas sensing applications. ZnO is an n-type II–VI semiconductor with a direct band gap of approximately 3.37 eV at room temperature and a relatively high exciton binding energy of about 60 meV. Its high electron mobility, along with strong thermal and chemical stability, makes it particularly suitable for sensing applications.^38^ In addition, ZnO-based sensors are widely used for detecting flammable and toxic gases due to their non-toxicity, low cost, and ease of fabrication.^39^

Recent technological advancements have significantly improved the fabrication methods used for ZnO-based gas sensors, enabling better control over their structural and morphological properties.^40^ The sensing performance of ZnO can be further enhanced through strategies such as elemental doping and the formation of composite materials.^41^ Zinc oxide nanoparticles (ZnO NPs) also exhibit notable antibacterial properties, which enable their use as effective antimicrobial agents in various applications.^42^ These advantages make ZnO nanoparticles attractive candidates for developing sensitive and selective gas sensing devices.

ZnO nanoparticles can be synthesized using a variety of chemical and physical methods. Common synthesis approaches include homogeneous precipitation, direct precipitation, the sonochemical method, solvothermal synthesis, the sol–gel method, reverse micelle synthesis, microwave irradiation, and thermal decomposition.^23^ Each of these methods provides distinct advantages in controlling particle size, morphology, crystallinity, and surface properties, which significantly influence the sensing performance of ZnO-based materials.

Despite the development of several ammonia sensing technologies, many existing systems rely on expensive materials, complex fabrication procedures, and sophisticated instrumentation, which limit their large-scale practical application. Therefore, there is an increasing demand for cost-effective, sustainable, and easily fabricated sensing materials. Natural pigments such as anthocyanins have recently attracted significant attention due to their pH-sensitive colorimetric properties, antioxidant activity, environmental friendliness, and renewable nature. Anthocyanins, which are abundant in various fruits and vegetables, exhibit unique optical characteristics that make them suitable for sensing applications.

The integration of anthocyanins with metal oxide semiconductors such as ZnO offers a promising strategy for developing efficient sensing platforms.^43^ ZnO nanoparticles provide a high surface area, strong chemical stability, and excellent sensitivity toward gaseous analytes, which can enhance the performance of anthocyanin-based sensing systems. The combination of these materials may therefore provide a synergistic platform for ammonia detection associated with food spoilage.

In this context, the present study aims to fabricate and characterize an anthocyanin-loaded ZnO sensing system and evaluate its potential for ammonia detection as an indicator of food spoilage. The structural, morphological, and sensing properties of the developed material are systematically investigated to assess its suitability as a low-cost and environmentally friendly food freshness monitoring platform. In this study we focus on the optical detection of ammonia in aqueous solutions (liquid phase), and does not address gas-phase ammonia sensing.

## 2. Materials and methods

### 2.1 Chemicals

Zinc acetate (Zn (CH_3_COO)_2_·2H_2_O) from Merk, India. Hydrochloric acid (HCl) Sodium Hydroxide (NaOH) and Ammonia from CDH India Limited, Ethanol from Merk, India. Distilled water.

### 2.2 Collection of plant material and preparation of plant specimens

Bottlebrush (Melaleuca) flowers were collected from the Indira Gandhi National Tribal University Amarkantak (IGNTU) (Latitude 22.80246° north, Longitude 81.75114° east) Madhya Pradesh, India during May 2023. The collection of plant material was performed by following the national and international guidelines.^44^ The plant specimens were identified by subject expert Dr Ravindra Shukla from the Department of Botany and voucher specimens were deposited in the Department of Botany, IGNTU Amarkantak, MP (India) with voucher number (IGNTU/DOB/2024/myr/mc/04). The flowers, stems and leaves were used in this investigation. The specimens were washed carefully with distilled water twice to remove impurities and dirt and further kept for drying for 2 weeks. After drying, the specimens were grounded to a fine powder with mortar and pestle and then preserved in airtight jars for later use.

### 2.3 Preparation of plant extract

Five grams of flower powder was taken in 100 ml of distilled water and heated at 70 °C for half an hour. The extract was kept for cooling at room temperature. The pH of the flower extract was 6.48. The flower extract was filtered by using Whatman filter paper No. 1. A natural light reddish color solution was obtained and stored at 4 °C for further use in the experiment.

### 2.4 Preparation of ZnO nanoparticles

In brief, 0.01 M zinc acetate dihydrate (Zn (CH_3_COO)_2_·2H_2_O) solution was prepared in double distilled water. For the synthesis of zinc oxide nanoparticles, 8 ml plant extract was added to 92 ml of 0.01 M zinc acetate dihydrate and thus the solution color was changed to light green color. The pH of the mixture was 6.11, 1 M solution of NaOH was added dropwise till pH = 9 and a gelatinous white-greenish precipitate was obtained. The mixture was stirred for 3 hours at 80 °C. The resultant light green solution was centrifuged and washed thrice with water and twice with ethanol. The obtained precipitate was dried at 70 °C in an oven and kept overnight to yield powdered zinc oxide nanoparticles.^45–48^ The synthesized nanoparticle using green method was then used an ammonia gas sensor and measured by UV-Vis spectrophotometer.

### 2.5 Fabrication of paper sensor with deposition of ZnO NPs and bottlebrush flower anthocyanin

The paper-based sensor was prepared by using Whatman filter paper 42 by cutting 2 cm × 2 cm area of rectangular shape and a 20 µL of ZnO NPs was deposited on the filter paper and dried at room temperature and then dipped in 1 ml of bottlebrush flower extract.

### 2.6 Image capturing

Digital microscope camera was used to capture the images of color developed after sensing of ammonia gas. The paper was placed perpendicular to the microscope with a distance of 20 cm and paper strip was enlightened with constant LED flash light source. The captured images from microscope was imported to the Image J software (National Institute of Health, USA) for determination of mean color intensity of developed paper-based sensor. The mean pixel intensity was obtained by considering RGB color model and Lab (*L*: Lightness, *a*: Redness, *b*: Yellowness) value.

### 2.7 Characterization of biosynthesized ZnO-NPs

To evaluate the physical and chemical characteristics of zinc oxide nanoparticles (ZnO-NPs) synthesized using bottlebrush flower extract, a variety of analytical techniques were employed. These included UV-visible spectroscopies, Fourier-transform infrared (FTIR) spectroscopy, X-ray diffraction (XRD), transmission electron microscopy (TEM), and dynamic light scattering (DLS). The interaction between the plant extract and zinc acetate dihydrate solution was studied using a Shimadzu UV-1800 spectrophotometer, scanning wavelengths from 200 to 700 nm. XRD analysis was carried out using a PANalytical X'Pert3 diffractometer to determine the crystalline structure of the synthesized nanoparticles. Crystallite size was estimated *via* the Scherrer equation.^49^

1 *D* = *kλ*/*β* cos *θ*

where *D* is the average crystallite dimension, *k* is a shape factor (0.94), *λ* represents the X-ray wavelength (1.5421 Å), *β* is the full width at half maximum (FWHM) in radians, and *θ* is the Bragg angle.

FTIR analysis (Jasco FTIR-4600) was conducted over a spectral range of 400–4000 cm^−1^ to identify the functional groups involved in nanoparticle formation. Particle shape and size were visualized using a high-resolution transmission electron microscope (TALOS, Thermo Scientific, 100 kV, AIIMS Delhi). DLS studies were carried out using a Malvern Zetasizer (Anton Paar Litesizer 500) with a 12 mm cuvette to determine the size distribution and *ζ*-potential. The *ζ*-potential, indicating the stability and surface charge behavior of the colloidal particles, was measured in phase analysis light scattering mode over 100 cycles under stable room temperature conditions. It was calculated using the Smoluchowski equation:

2 *v* = (*εξ*/*η*)*E*

where *v* is the electrophoretic mobility, *ε* the dielectric constant, *ξ* the zeta potential, *η* the viscosity of the medium, and *E* the applied electric field.

### 2.8 Sensing study of ammonia and carbon dioxide detection

In the context of the sensing study, a solution containing 25% ammonia is utilized. The solutions with varying concentrations ranging from 0 ppm, 5 ppm, 10 ppm, 20 ppm, 40 ppm, 60 ppm, 80 ppm and 100 ppm were prepared using distilled water immediately before the experiment.^50^ For selectivity measurements of ammonia and carbon dioxide, the preparation of both gases was conducted following previously reported procedures.^51,52^

### 2.9 Antimicrobial study and isolation of bacterial pathogens

The antimicrobial study was conducted in the Infection Biology and Molecular Reproductive Toxicology Lab, Department of Zoology, IGNTU, Amarkantak, Madhya Pradesh, India. *E. coli* and *K. pneumoniae* involved in this study were isolated from clinical samples. Whereas the MTCC strain of *S. aureus* and *L. Monocytogenes* were purchased from the Institute of Microbial Technology (IMTECH), Chandigarh.

### 2.10 Antibiotic sensitivity testing (AST)

The antibiotic sensitivity tests (ASTs) on the selected bacterial isolates were performed using the agar well-diffusion and disk diffusion method as described previously by Sharma and Cowerkers.^53^

### 2.11 Study of antioxidant activity

The antioxidant activity of bio-synthesized ZnO nanoparticles was measured by the DPPH method.^54,55^ 2 µg of DPPH was dissolved in ethanol in a 100 ml volumetric flask. ZnO nanoparticles with various concentrations 50 µg, 100 µg, 200 µg, 400 µg and 800 µg was dispersed in DPPH solution.

Free radicals produced by DPPH, were scavenged by ZnO nanoparticles. Thus, the initial deep violet color of solution due to DPPH radical gradually turned to colourless or light yellow when ZnO NPs were present. This change in solution color affects the percentage of absorption at 518 nm, is used to track the concentration of radicals.

DPPH scavenging activity is calculated using the equation:

3 DPPH scavenging activity (%) = (*A*_c_ − *A*_s_/*A*_c_) × 100

where *A*_c_ is the absorbance at 518 nm for control (DPPH) and *A*_s_ is the absorbance of the sample (various concentrations of ZnO nanoparticles in DPPH solution).

## 3. Results and discussion

### 3.1 UV-visible spectroscopy

**Fig. 1:**
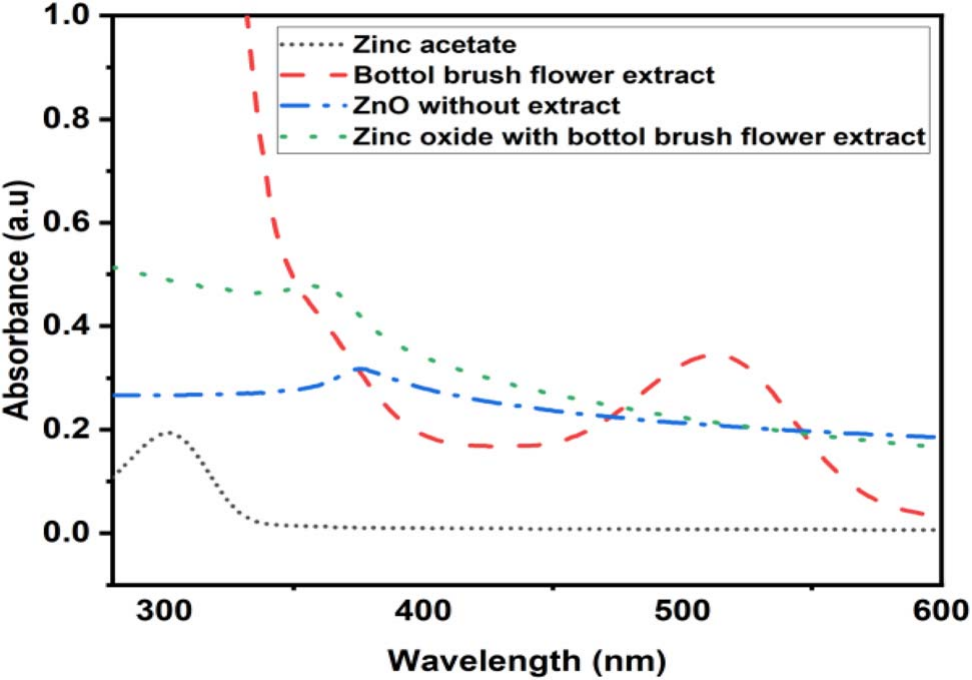
UV-visible spectra of bio-synthesized ZnO (with flower extract) NPs, bottlebrush flower extract and zinc acetate.

**Fig. 2:**
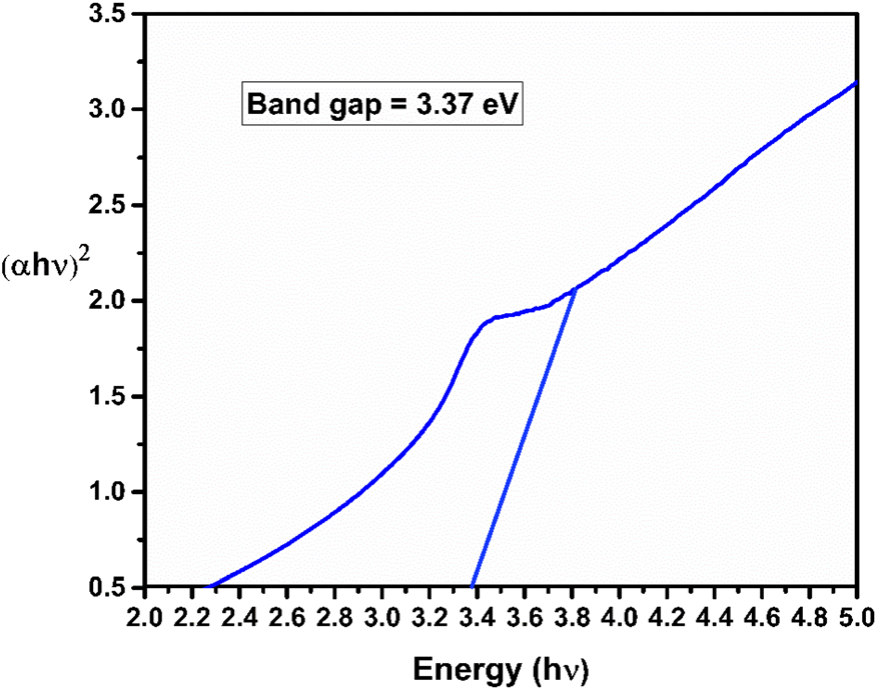
The band gap of bio-synthesized ZnO nanoparticles.

The spectra in Fig. 1 showed sharp peak characteristics of bio-synthesized ZnO nanoparticles at 383 nm and the peak appered at 385 nm is corresponding to ZnO nanoparticle without bottlebrush flower extract. Further, the peak at 538 nm and 307 nm confirms the anthocyanin and zinc acetate respectively. The optical bandgap energy of biosynthesized ZnO NPs was calculated by using tauc formula and the bandgap energy was found 3.37 eV at wavelength of 368 nm which is shown in Fig. 2.^56,57^ The calculated value of bandgap energy of biosynthesized ZnO NPs were in good agreement with the optical bandgap energy of standard ZnO nanoparticles.

To calculate the energy band gap we used the Tauc formula:

4 (*Ahv*)*γ* = *Aα*(*hv* − *E*_g_)

where *E*_g_ denotes energy bandgap and *h* is planks constant, *α* shows absorption coefficient, *v* is the frequency which is given by (*v*= *c*/*λ*) and *γ* denotes nature of electronic transition which is equal to 1/2 for direct bandgap, *A* is constant, usually taken as 1 for amorphous materials.

### 3.2 FT-IR analysis

**Fig. 3:**
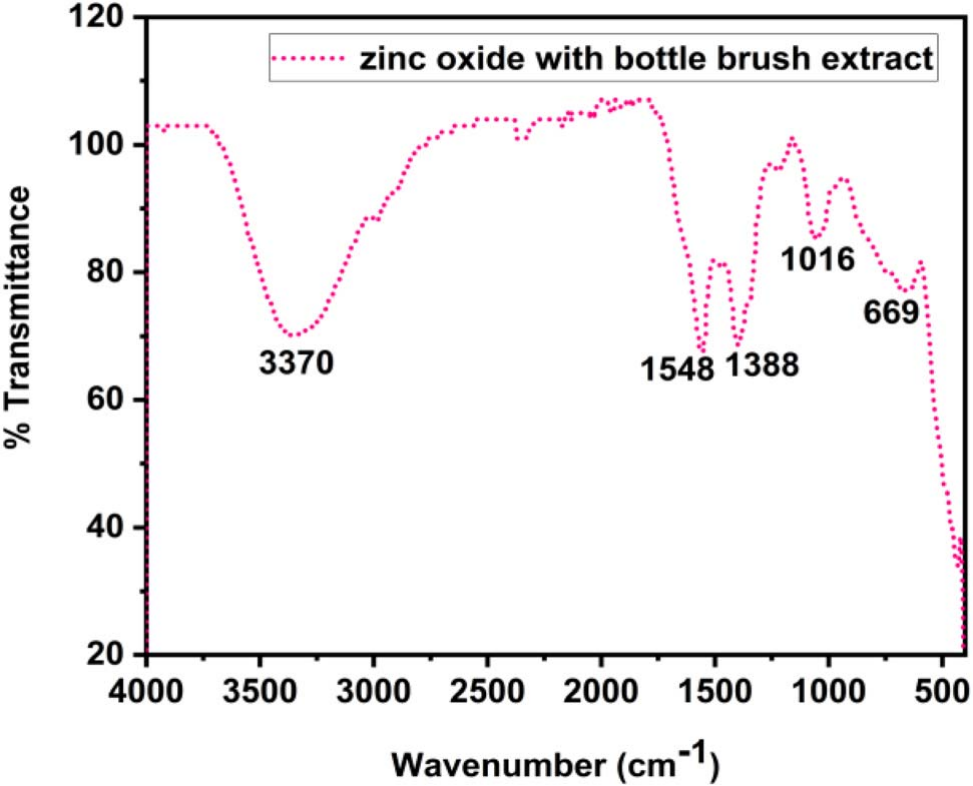
FT-IR spectra of bio-synthesized ZnO nanoparticles with bottlebrush flower extract.

Synthesized zinc oxide nanoparticles were subjected to FT-IR analysis to identify the different characteristic functional group along with the synthesized ZnO nanoparticles. FT-IR spectrum of the synthesized ZnO nanoparticles showed in Fig. 3. The peaks in the spectra indicate the characteristics functional group found in the synthesized zinc oxide nanoparticles. The various peaks are 3370 cm^−1^, 1548 cm^−1^, 1388 cm^−1^, 1016 cm^−1^ and 669 cm^−1^. The peak at 669 cm^−1^ corresponds to metal–oxygen (ZnO stretching vibrations) vibration mode.^58^ The peak at 1016 cm^−1^ is described to the stretching vibration of C–N bond of the primary amine. The peak at 1388 cm^−1^ and 1548 cm^−1^ corresponds to primary or secondary alcohol in plane, bend or vibration. The peak at 3370 cm^−1^ corresponds to the stretching vibration of hydroxyl compounds. These results are supported by other studies.^42,59^ The results indicate that both the hydroxyl groups present in flavonoids and the functional groups of proteins contribute significantly to the reduction of metal salts and the stabilization of nanoparticles.

### 3.3 X-ray diffraction (XRD)

**Fig. 4:**
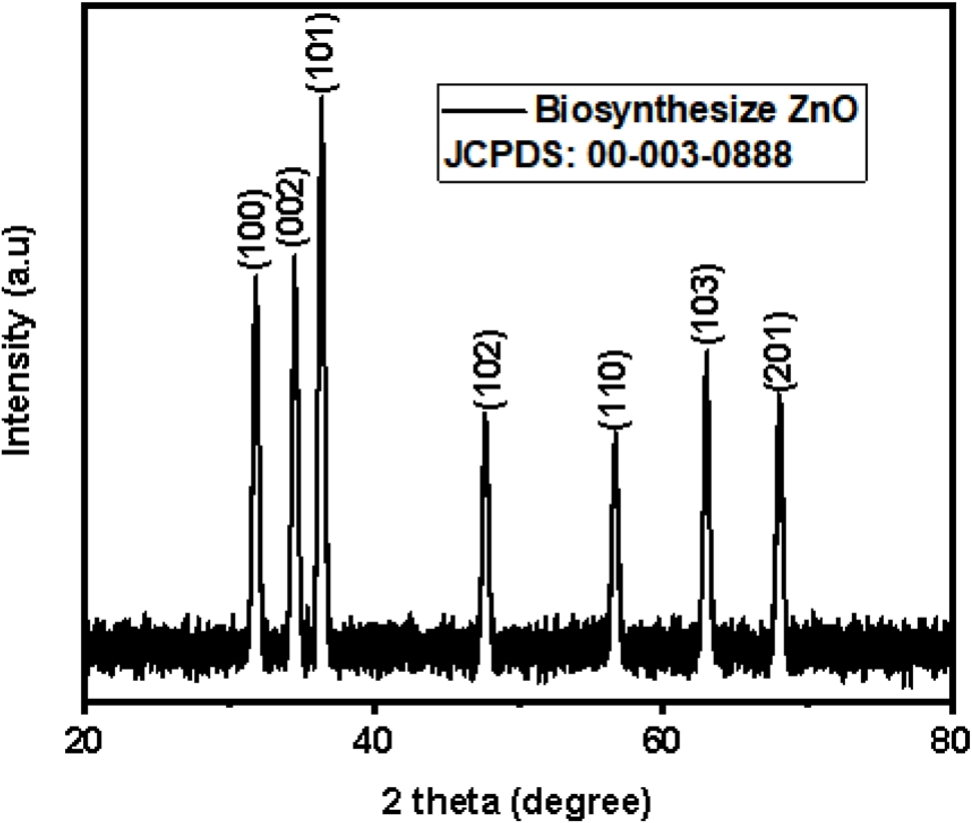
XRD pattern of bio-synthesized ZnO nanoparticles.

**Fig. 5:**
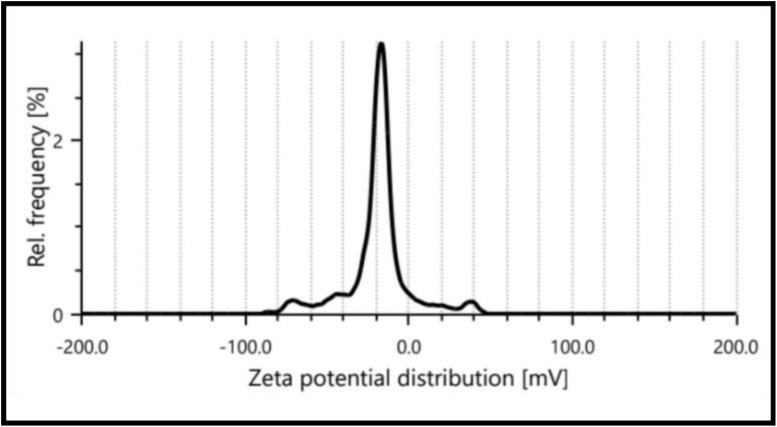
Zeta potential of the biosynthesized ZnO nanoparticle.

X-ray diffraction was used to detect the crystalline phase of biosynthesized ZnO nanoparticles. The Bragg's reflection plane in the 2*θ* range between 30–70° showed by XRD pattern. Fig. 4 represented the diffraction peaks at 2*θ* = 31.8°, 34.5°, 36.3°, 47.6°, 56.7°, 62.9° and 67.8° corresponds to the *h*, *k*, *l* plane. These peaks are similar to other reported studies.^60,61^ This XRD pattern matches the phase of standard ZnO (JSPDS code 00-003-0888) which is a hexagonal wurtzite polycrystalline structure with lattice planes (hkl) of (100), (002), (101), (102), (110), (103) and (112), as illustrated in Fig. 5. By using Scherrer formula, average crystalline size is found to be 28.56 nm. The sharp and narrow peaks of the XRD pattern confirmed the crystalline nature of biosynthesized ZnO nanoparticle.^62^

### 3.4 Zeta potential analysis

Zeta potential analysis assesses the surface charge of ZnO nanoparticles in solution. When ZnO nanoparticles are dispersed in a liquid medium, they acquire a surface charge due to ion adsorption. The Zeta potential represents the electrical potential difference between the nanoparticle surfaces and the surrounding liquid medium. It reflects the stability of the nanoparticle dispersion, as particles with high zeta potentials repel each other, preventing aggregation. As shown in Fig. 5, the biosynthesized ZnO nanoparticle showed a mean zeta potential of −23.4 mV indicating that the prepared nanoparticles are moderately stable. The capping of biomolecules present on the biosynthesized nanoparticles mostly consisted of a negatively charged group was confirmed by this result.^63^ The detected negative charge of the synthesized ZnO nanoparticle revealed the electrostatic repulsion between nanoparticles.

### 3.5 Dynamic light scattering (DLS) analysis

**Fig. 6:**
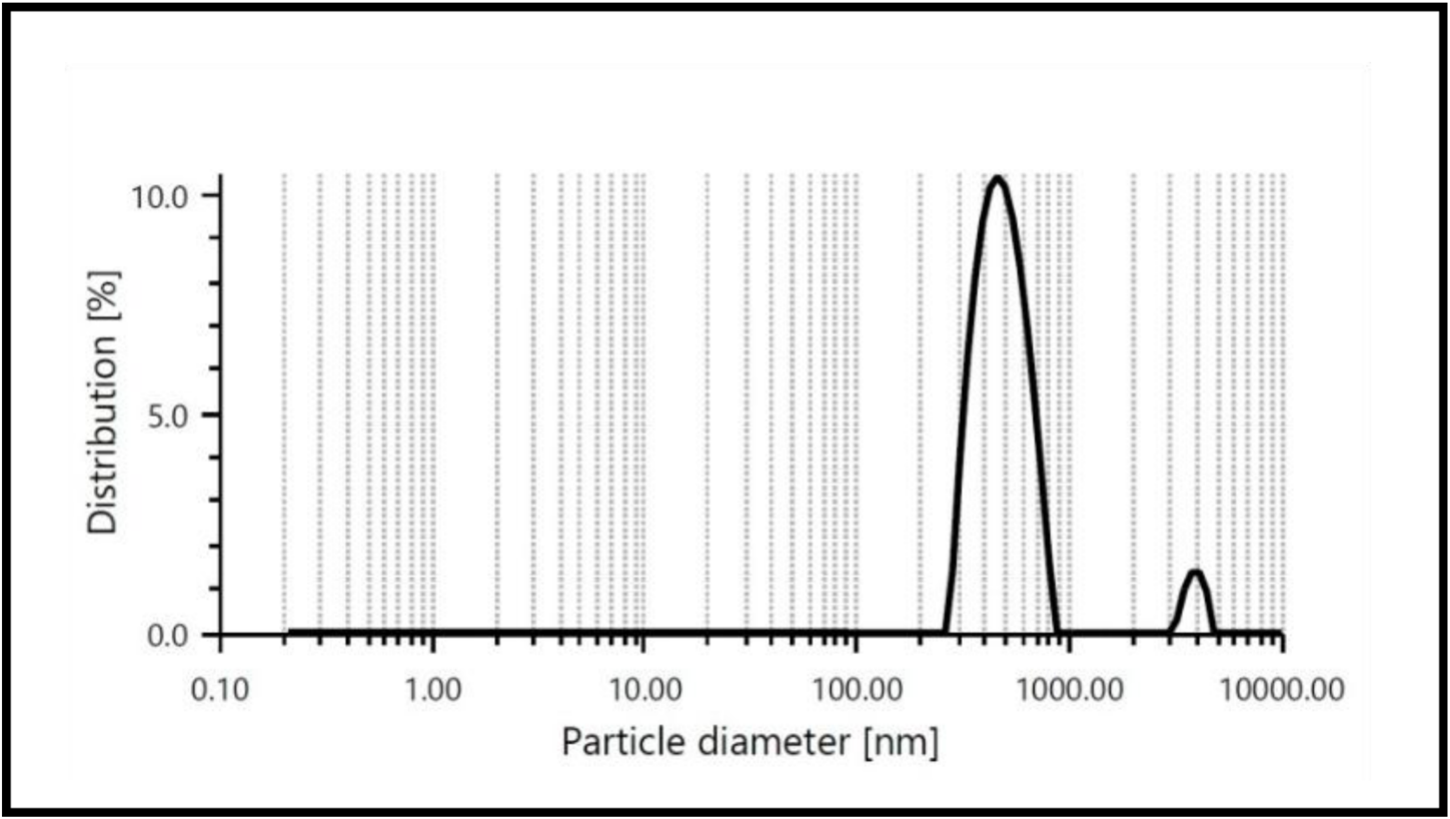
DLS analysis of bio-synthesized ZnO nanoparticle.

Dynamic light scattering (DLS) analysis is utilized to determine the hydrodynamic diameter of zinc oxide nanoparticles in suspension. When zinc oxide nanoparticles are dispersed in a liquid medium, they undergo Brownian motion, causing fluctuations in the intensity of scattered light. DLS measures these fluctuations to calculate the diffusion coefficient, which is then used to determine the hydrodynamic diameter of the nanoparticles through the Stokes–Einstein equation. The average hydrodynamic particle size of ZnO shown in Fig. 6 as calculated by DLS was found quite larger than that detected by TEM analysis and the theoretical size of the nanoparticles was calculated by using XRD. The polydispersity (PDI) values shows the variation in nanoparticle size as agglomerates or aggregates. The PDI of ZnO was found 0.191, thus the larger size of ZnO nanoparticles found in DLS as compared to TEM analysis due to the accumulation of extra hydrate layers on the nanoparticle surface.

### 3.6 Transmission electron microscopy (TEM) analysis

**Fig. 7:**
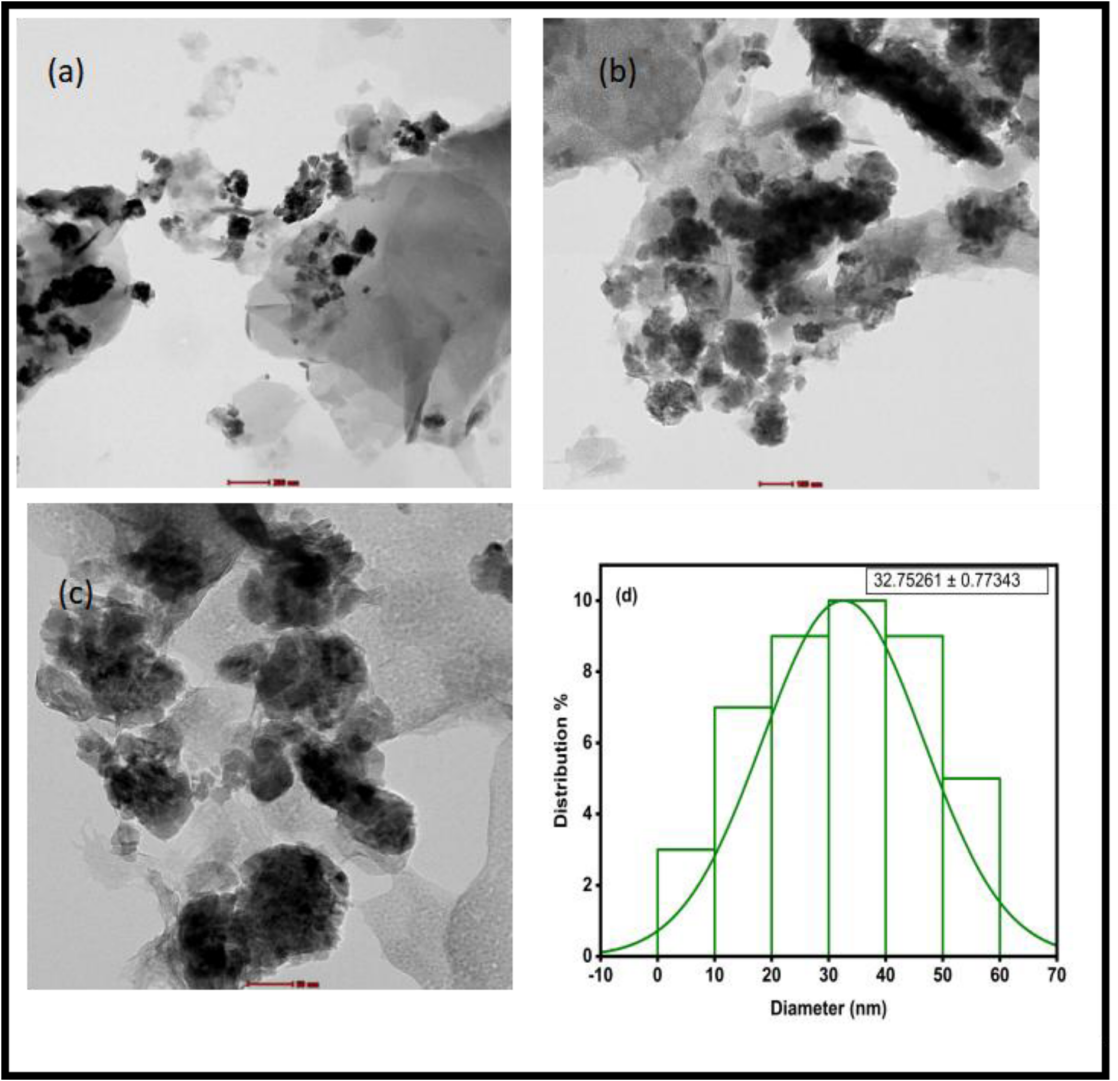
TEM images at different magnifications (a–c) and particle size distribution of bio-synthesized ZnO nanoparticles (d).

The morphological analysis and size of biosynthesized ZnO nanoparticles was performed by TEM analysis.^64^ The TEM images at different magnification (50, 100 and 200 nm) as shown in Fig. 7(a–c) indicated the formation of biosynthesized ZnO NPs. The images obtained from TEM revealed that the particles were agglomerated and irregular in shape (roughly spherical and cubical in shape). The average particle size distribution indicated that the average particle size diameter of ZnO NPs was found 32.75 ± 0.77 nm which is shown in Fig. 7(d). These results are in good agreement with Wali Muhammad *et al.* (2019),^65^ where the green synthesis of ZnO NPs by using Papaversomniferum L. with irregular and spherical shapes are confirmed. These results are in good agreement with the XRD data.

### 3.7 Ammonia sensing performance analysis

**Fig. 8:**
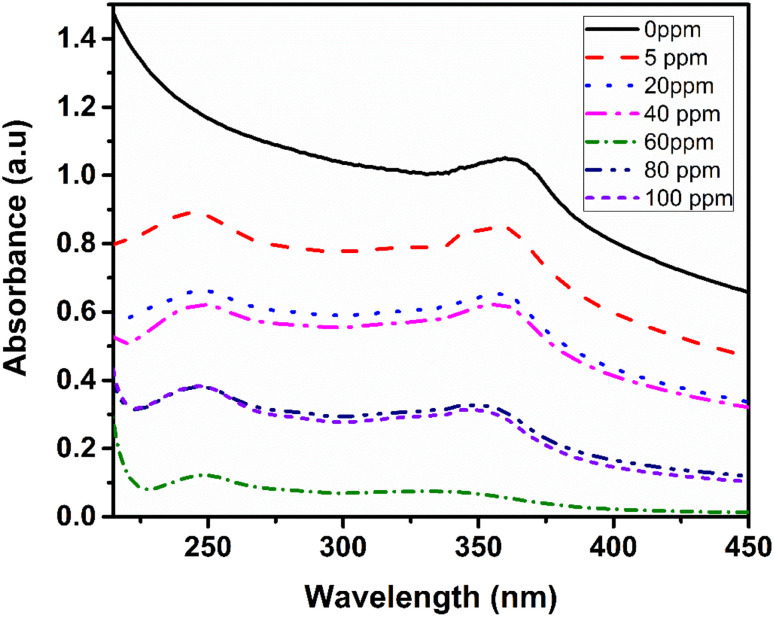
UV-vis spectral absorbance of bio-synthesised ZnO NPs as a function of various ammonia concentrations.

The green synthesis of zinc oxide nanoparticles using bottlebrush anthocyanin as a bio-template offers an innovative approach that combines environmentally friendly, bio-based nanoparticles with effective ammonia gas sensing capabilities. Anthocyanin, a natural pigment, plays an essential role in both the formation and functionalization of the nanoparticles. These ZnO nanoparticles exhibit a distinct interaction with ammonia gas, which was analyzed using UV spectrophotometry. The ammonia sensing study, illustrated in Fig. 8, involved optical measurements and UV spectra of ammonia solutions at varying concentrations. The absorbance spectra increased as ammonia concentrations ranged from 0 to 100 ppm. For each measurement, fresh colloidal solutions of ammonia and ZnO nanoparticles were prepared. In the ammonia sensing test, 1 ml of ZnO nanoparticle solution was mixed with 1 ml of ammonia solutions at concentrations of 0, 5, 10, 20, 40, 80, and 100 ppm, using a 25% ammonia aqueous solution diluted to the desired concentrations. It was observed that as the ammonia concentration increased, the LSPR peak intensity at 361 nm diminished, and a new peak emerged at 250 nm. Shifts in the LSPR band are often attributed to changes in the dielectric constant and variations in the distance between nanoparticles in the surrounding medium. The interaction between ammonia and the nanoparticles leads to the formation of coordination complexes. Furthermore, different ammonia concentrations were tested to identify the detection limit. Fig. 8 demonstrates the appearance of a new peak at 250 nm in the ammonia solution at 5 ppm, suggesting that 5 ppm may be the detection limit for this system. Consequently, for subsequent experiments, 5 ppm was selected as the lowest ammonia concentration. These results are consistent with previous studies.^50,62^

**Fig. 9:**
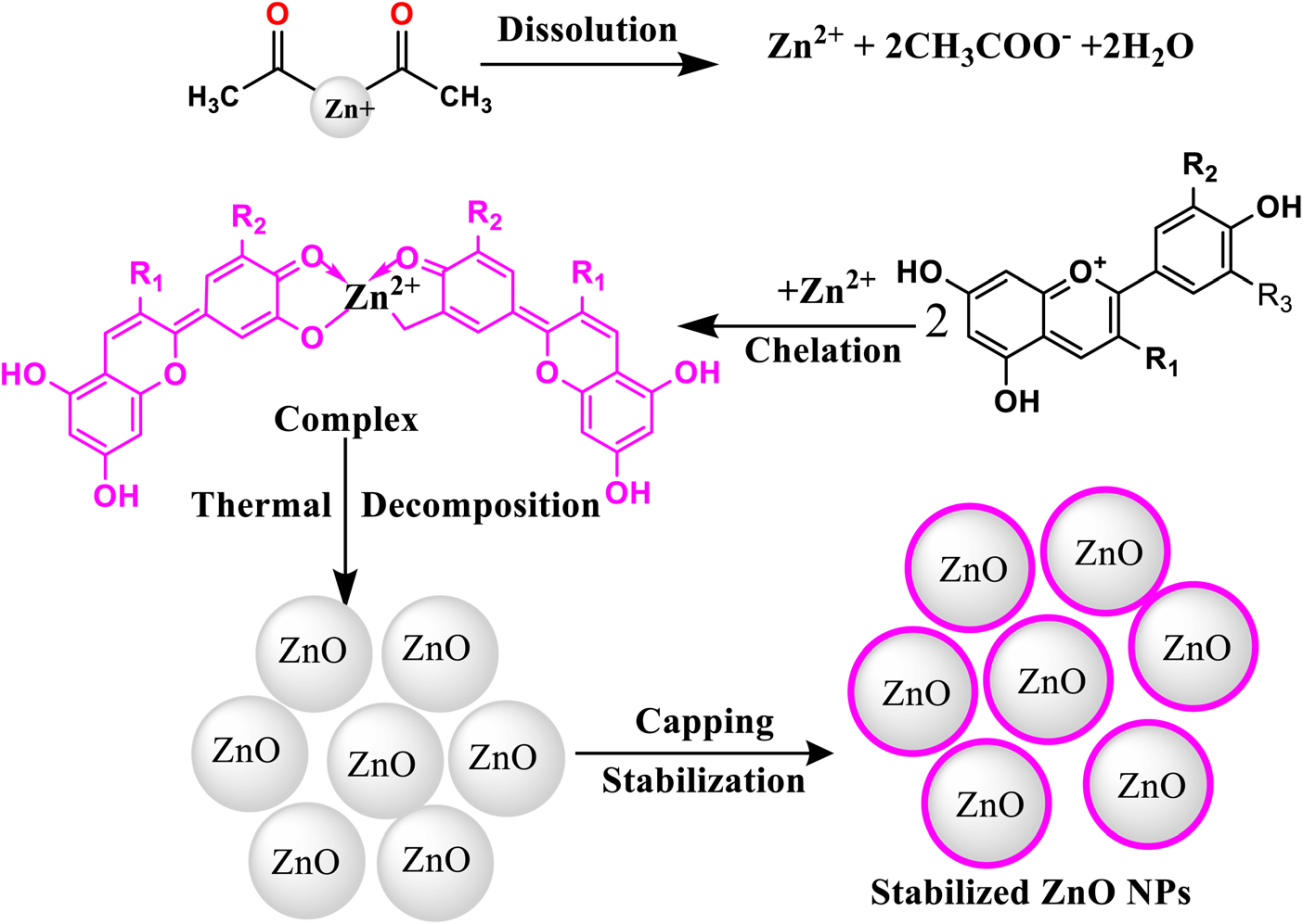
Green synthesis of ZnO nanoparticles using anthocyanin extract from *Callistemon citrinus* (bottlebrush), acting as a reducing and stabilizing agent.

**Fig. 10:**
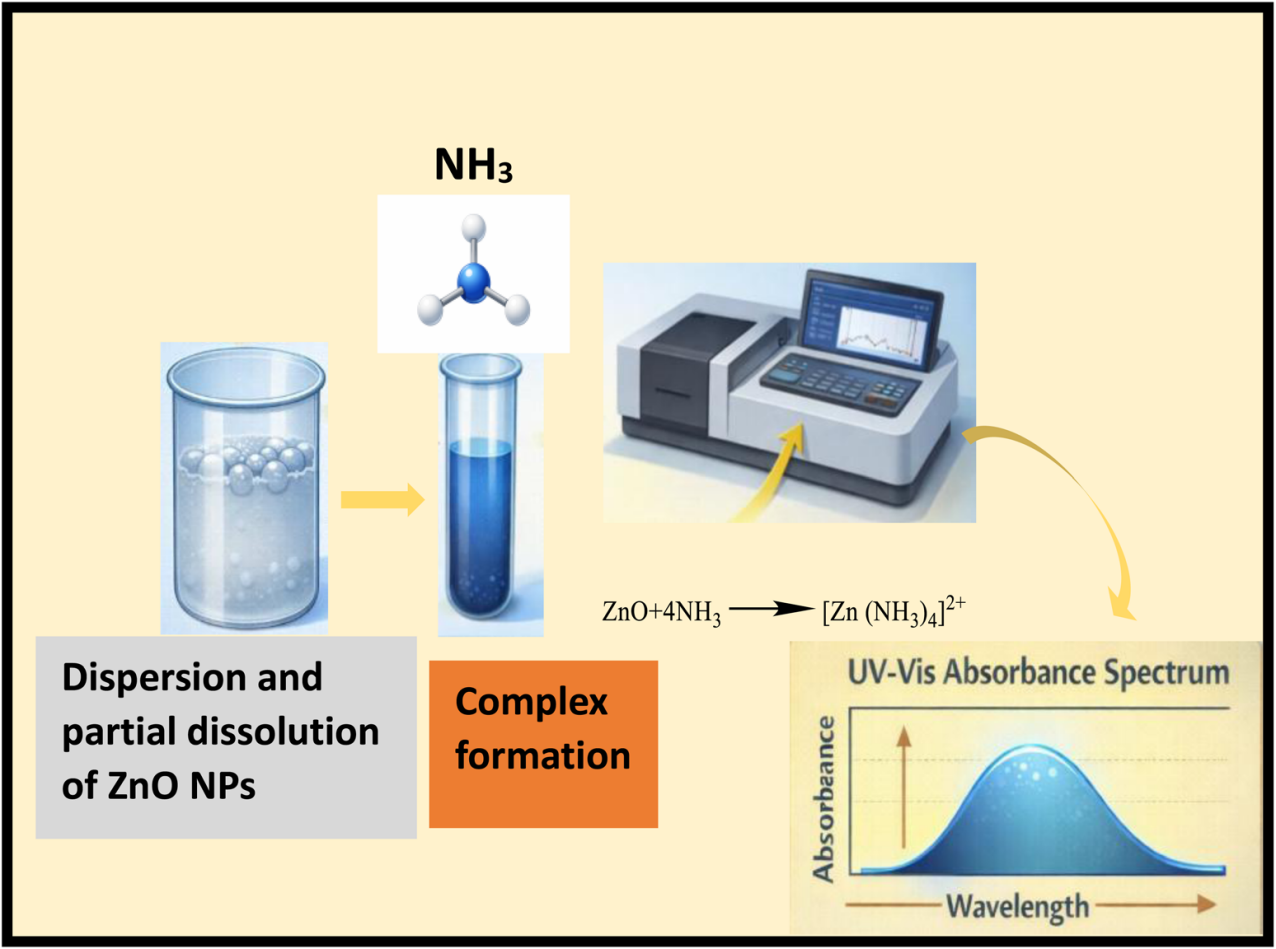
Schematic of ammonia sensing *via* reversible complex formation between NH_3_ and active sites, producing a measurable signal change.

The mechanism involved in ammonia sensing using biosynthesized ZnO NPs revolves around the interaction between ammonia gas and the ZnO nanoparticle surface, which might be stabilized by bottlebrush flower anthocyanin shown in Fig. 9. The ZnO nanoparticles biosynthesized using bottlebrush flower extract were employed for the optical detection of ammonia in aqueous medium shown in Fig. 10. In solution, ammonia exists in equilibrium between NH_3_ and NH_4_^+^, with NH_3_ acting as a Lewis base capable of coordinating to surface-exposed Zn^2+^ sites on ZnO nanoparticles. This interaction can be described as the formation of surface Zn–NH_3_ coordination complexes. While tetraammine zinc(ii) species, [Zn (NH_3_)_4_]^2+^, are well known in homogeneous aqueous systems containing free Zn^2+^ ions, in the present case the coordination is more appropriately considered as surface-bound complexation rather than the formation of fully solvated complexes.

The presence of anthocyanin-rich phytochemicals from the bottlebrush extract further modifies the ZnO surface, introducing functional groups that facilitate ammonia binding and stabilize intermediate coordination structures. These interactions alter the local electronic environment of the ZnO nanoparticles, particularly affecting surface states and band-edge characteristics.

Consequently, the formation of such coordination interactions leads to measurable changes in the UV-Vis absorption spectra, including variations in absorbance intensity and possible spectral shifts. The sensing mechanism is therefore attributed to solution-phase coordination of ammonia at the nanoparticle interface and the resulting modulation of optical properties, rather than gas-phase adsorption or bulk complex formation. This sensor not only allows for effective ammonia detection but also ensures sustainable and environmentally friendly synthesis route.

#### 3.7.1 Gas sensing performance toward ammonia and carbon dioxide

**Fig. 11:**
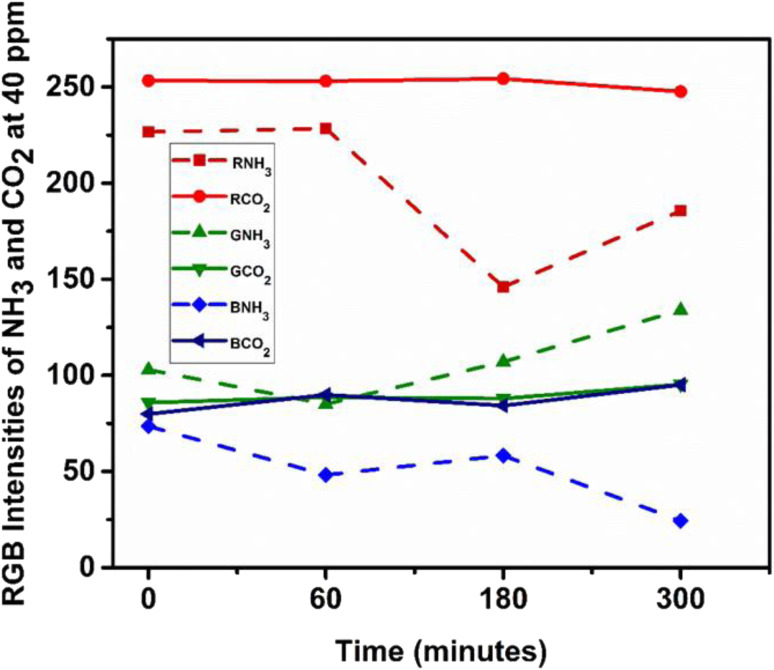
RGB analysis illustrating the selectivity and sensitivity of the sensor towards NH_3_ and CO_2_, based on distinct color intensity changes upon gas exposure.

The gas sensing performance of the ZnO-coated, bottlebrush anthocyanin-infused filter paper was evaluated toward NH_3_ and CO_2_ at 40 ppm under standard and high humidity (>90% RH) conditions. Time-dependent RGB analysis (Fig. 11) shows a pronounced color change for NH_3_, whereas CO_2_ induces minimal variation.

**Fig. 12:**
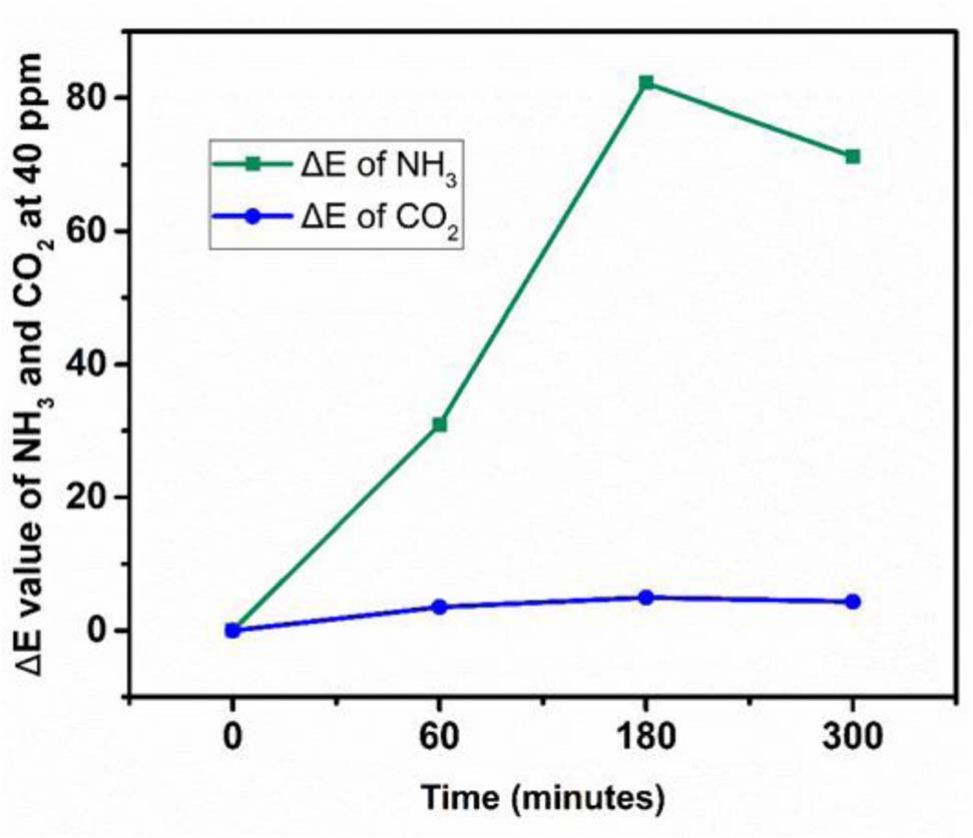
Δ*E* values showing sensor selectivity towards NH_3_ and CO_2_.

**Fig. 13:**
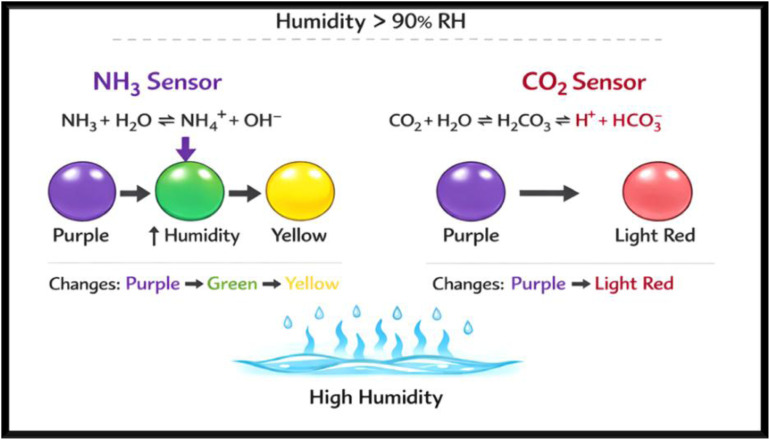
Selectivity mechanism for NH_3_ and CO_2_ under high humidity conditions.

The Δ*E* values for NH_3_ increased significantly from 60 to 180 min, followed by a slight decrease at 300 min, indicating rapid adsorption and subsequent saturation of active sites. In contrast, CO_2_ exhibited negligible Δ*E* variation, confirming weak interaction with the sensing layer. The selectivity factor (Δ*E*−NH_3_/Δ*E*−CO_2_) increased from ∼8.7 to ∼16.6, demonstrating excellent selectivity toward NH_3_ even under high humidity. The sensing mechanism (Fig. 12 and 13) is strongly influenced by moisture at >90% RH.

**Fig. 14:**
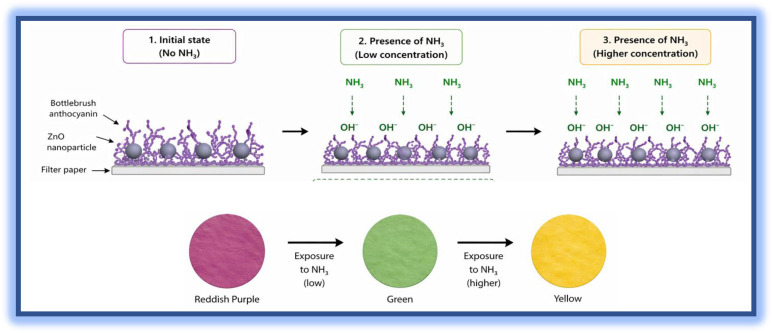
Schematic illustration of NH_3_ sensing by ZnO–anthocyanin coated paper, showing interaction-induced signal change.

Adsorbed water on the ZnO surface generates hydroxyl groups that enhance NH_3_ adsorption *via* hydrogen bonding and facilitate proton transfer to anthocyanin, leading to its deprotonation and a pronounced color change shown in Fig. 14. The slight decrease in Δ*E* at prolonged exposure (300 min) is attributed to site saturation and competitive adsorption with water. In contrast, CO_2_ forms weak carbonic acid in the presence of moisture, resulting in minimal pH change and negligible colorimetric response.

These results confirm that the ZnO-anthocyanin coated filter paper exhibits high sensitivity and excellent selectivity toward NH_3_ at 40 ppm, even under humid conditions, highlighting its suitability for practical sensing applications.

#### 3.7.2 Color stability of the sensor at different time interval

The colour stability of the paper sensor was evaluated after storage for 3 months at 60% relative humidity (RH). Stability was assessed by monitoring the sensor response during chicken spoilage at different storage times under ambient conditions. Colour changes were recorded using a digital microscope camera, and RGB values were extracted using ImageJ software and subsequently converted into CIELAB (L*, a*, b*) parameters for quantitative analysis. The stability of the sensor was determined by comparing the colour coordinates before and after storage, with the total colour difference (Δ*E*) used as a quantitative metric which is shown in Tables 1 and 2. The Δ*E* values indicated only minor variations between fresh and stored sensors, demonstrating that the sensor retained its functional colour response after 3 months storage. Furthermore, the consistent trends observed during spoilage analysis confirm that storage at 60% RH does not significantly affect sensor performance. These results are in agreement with the previously observed reproducibility of L*, a*, and b* values, supporting the reliability and practical applicability of the developed paper sensor.

**Table 1:** Initial stability performance of the fabricated sensor over repeated measurements and storage time (*n* = 3)

Color value ± sd	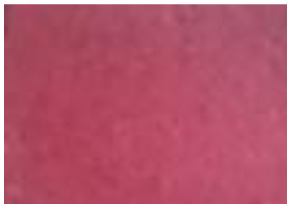	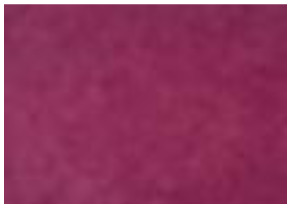	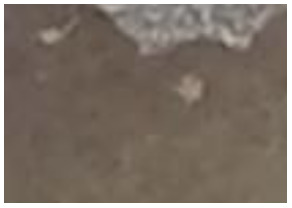	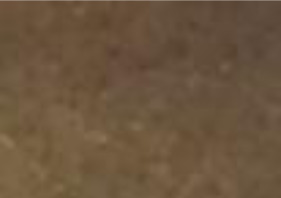
*L*	46.814 ± 0.49	33.642 ± 0.374	44.378 ± 0.486	37.910 ± 0.127
*a*	43.434 ± 0.514	41.959 ± 0.899	2.946 ± 0.114	5.313 ± 0.065
*b*	6.919 ± 0.511	−2.539 ± 0.289	8.037 ± 0.144	16.902 ± 0.308
Δ*E*	0	16.29	40.60	40.40

**Table 2:** Stability performance of the fabricated sensor after 3 months over repeated measurements and storage time (*n* = 3)

Color value	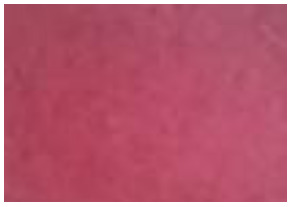	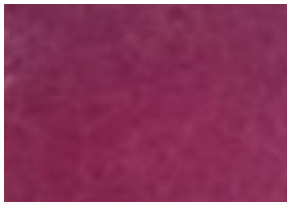	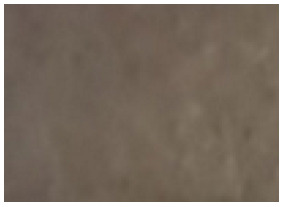	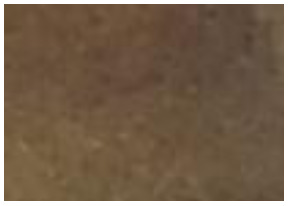
*L*	46.701 ± 0.076	33.212 ± 0.145	43.189 ± 1.123	38.074 ± 0.065
*a*	43.124 ± 0.279	41.696 ± 0.132	3.083 ± 0.153	5.513 ± 0.071
*b*	6.783 ± 0.132	−3.035 ± 0.107	9.064 ± 0.142	17.524 ± 0.059
	0	16.75	40.26	40.05

### 3.8 Antibacterial studies

**Fig. 15:**
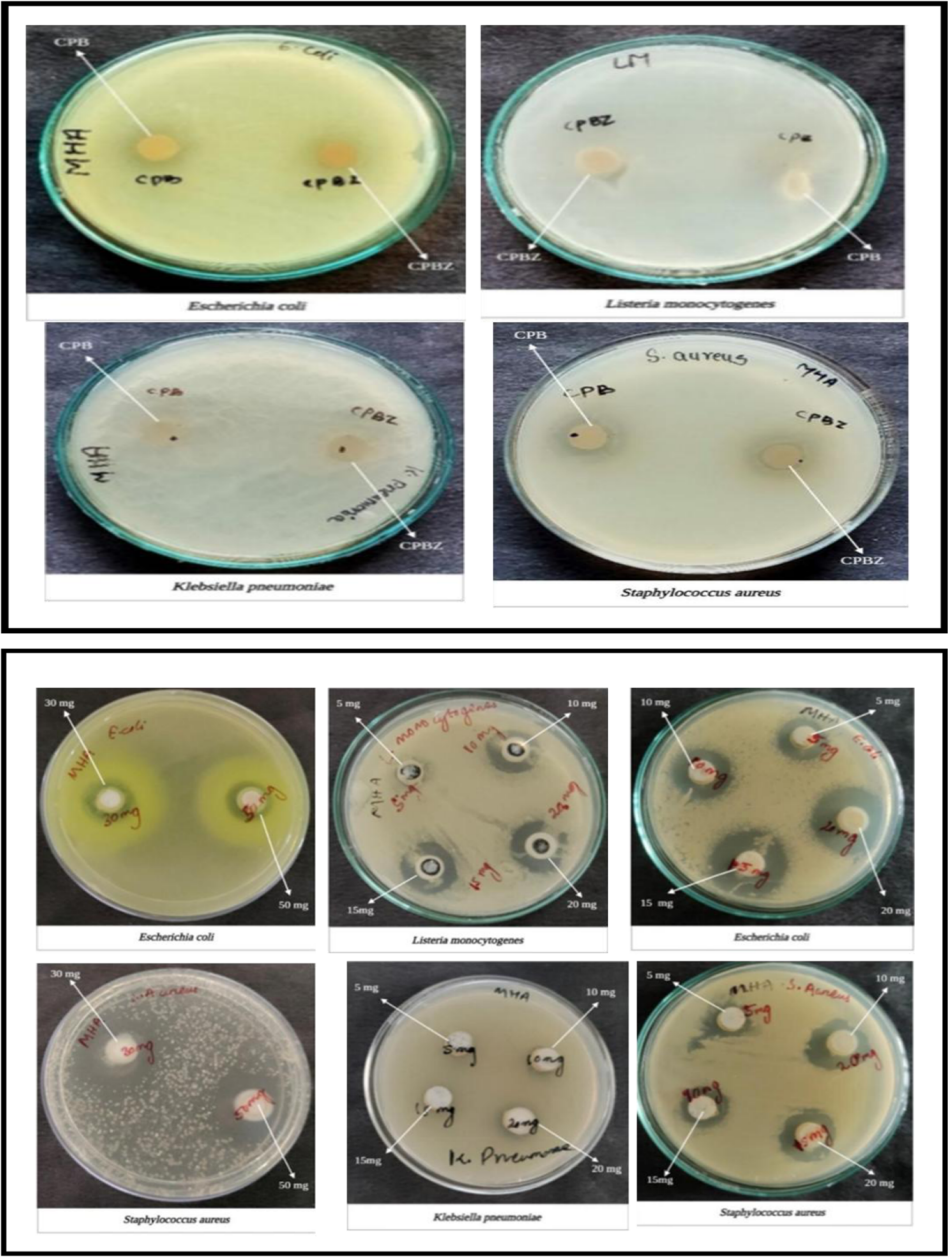
Concentration-dependent antimicrobial activities of nanomaterials against *L. monocytogenes*, *K. pneumoniae, E. coli*, and *S. aureus*. Cpbz: cellulose paper with bottlebrush extract and zinc oxide nanoparticles. Cpb: cellulose paper with bottlebrush extract.

Table 3 and Fig. 15 present data on the inhibition zones of various pathogens when exposed to different concentrations of nanomaterials. The inhibition zones are measured in millimeters (mm) and represent the effectiveness of the nanomaterials in inhibiting bacterial growth at six different concentrations: A (5 mg), B (10 mg), C (15 mg), D (20 mg), *E* (30 mg), and F (50 mg). The pathogens tested include *E. coli*, *K. pneumoniae*, *S. aureus*, and *L. monocytogenes*. *E. coli* showed its most effectivity at 20 mg, and *K. Pneumonia requires a* higher concentration (20 mg) for effectiveness. *S. aureus* effectiveness increases consistently with concentration, whereas *L. monocytogenes* effect starts from 10 mg, with slight increases at higher doses. Additionally, the fabricated sensor exhibited antimicrobial activity against *E. coli* and *S. aureus*, whereas *L. monocytogenes* and *K. pneumoniae* showed no inhibition zones.

**Table 3:** Inhibition zones of nanomaterials against various pathogens at different concentrations

Pathogens	Inhibition zones of nanomaterials at different concentrations					
	A (5 mg)	B (10 mg)	C (15 mg)	D (20 mg)	E (30 mg)	F (50 mg)
*E. coli*	12 mm	15 mm	19 mm	22 mm	14 mm	14 mm
*K. pneumoniae*	NZ	NZ	NZ	11 mm	—	—
*S. aureus*	11 mm	13 mm	15 mm	17 mm	22 mm	23 mm
*L. monocytogenes*	NZ	15 mm	16 mm	17 mm	—	—

### 3.9 Antioxidant activity

**Fig. 16:**
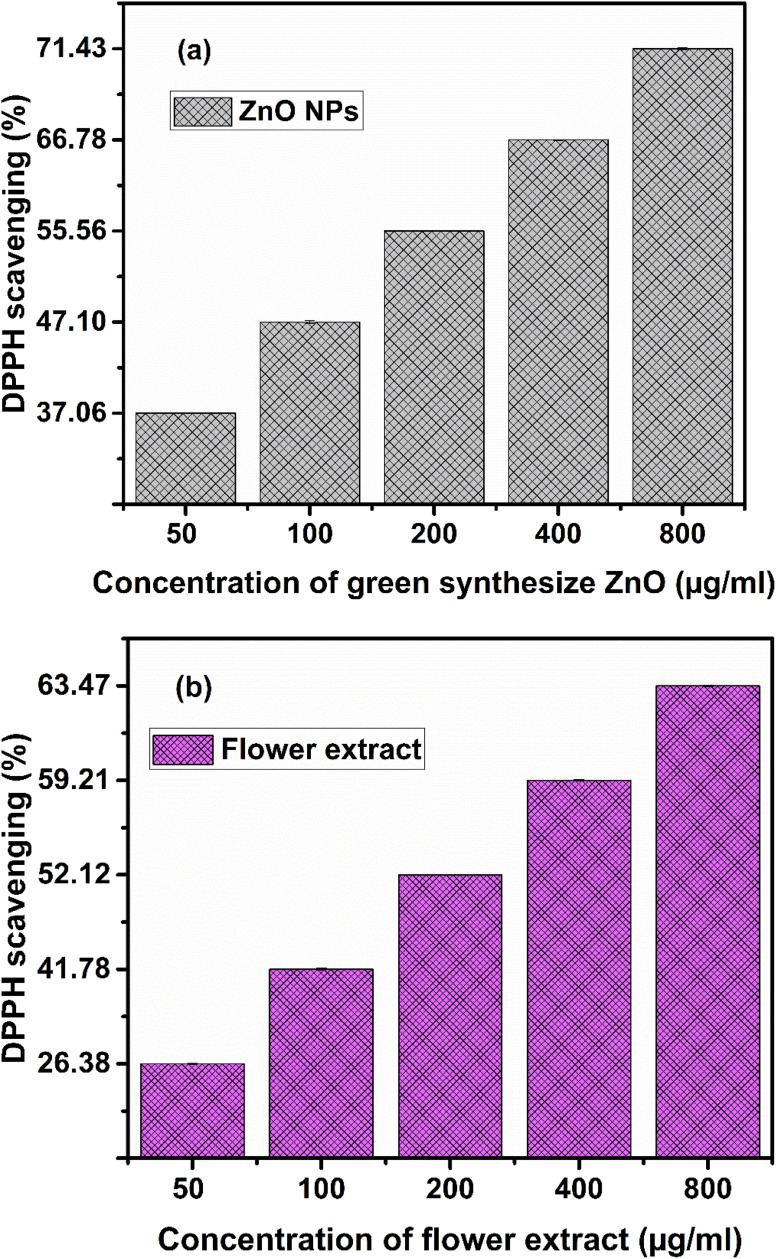
Radical scavenging activity of green-synthesised ZnO nanoparticles (a) and bottlebrush flower extract (b) evaluated using the DPPH assay at different concentrations (50–800 µg ml^−1^). Both samples exhibit a concentration-dependent increase in antioxidant activity; however, ZnO nanoparticles show consistently higher scavenging efficiency compared to the plant extract across all concentrations. Data are presented as mean ± standard deviation (*n* = 3).

The DPPH solution was left undisturbed for 1 hour in order to check its stability. Both the colour and the intensity of the absorption at 518 nm remain unchanged. This proves that the DPPH solution was steady throughout the experiment. Both the system and the nitrogen atom's unpaired electron exist in the DPPH. Molar absorptivity increases by substitution on aromatic rings, and this result enhances if the substitution lengthens the conjugation. The benzene absorption bands usually move from shorter to longer wavelengths as a result of the prolonged conjugation. The peak at 518 nm is due to n to π* transition. In the presence of ZnO nanoparticles, the colour of the DPPH solution progressively shifts from deep violet to pale yellow. The antioxidant activity of the samples was evaluated using the DPPH radical scavenging assay, and the results are presented in Fig. 16 a and b. A clear concentration-dependent increase in scavenging activity was observed for both the ZnO nanoparticles and the bottlebrush flower extract. The green-synthesised ZnO nanoparticles exhibited scavenging efficiencies of 37.06 ± 0.004%, 47.10 ± 0.029%, 55.56 ± 0.01%, 66.78 ± 0.01%, and 71.43 ± 0.023% at concentrations of 50, 100, 200, 400, and 800 µg ml^−1^, respectively. In contrast, the bottlebrush flower extract showed comparatively lower activities of 26.38 ± 0.01%, 41.78 ± 0.25%, 52.12 ± 0.003%, 59.21 ± 0.017%, and 63.47 ± 0.015% at the corresponding concentrations.

Notably, the ZnO nanoparticles demonstrated consistently higher scavenging activity across all tested concentrations, suggesting that the green synthesis process enhances their antioxidant potential. The enhanced antioxidant performance of the nanoparticles can be attributed to their high surface area-to-volume ratio and the presence of phytochemical residues from the extract acting as stabilising and functionalising agents, which may facilitate efficient electron or hydrogen donation to neutralise DPPH radicals.

These findings demonstrate that green-synthesised ZnO nanoparticles possess superior antioxidant activity compared to the crude bottlebrush flower extract, highlighting their potential for applications in antioxidant-related biomedical and packaging systems.

The electron density transfer from the oxygen to the nitrogen atom in DPPH, which results in a drop in the intensity of the n to π* transition at 518 nm, may be the cause of the antioxidant action of ZnO nanoparticles. Our results are showing good antioxidant activity in microgram range as compared to Dhaneswar das *et al.*^66^

### 3.10 Reliability of the sensor and analytical performance

The reliability of the developed sensor was confirmed through rigorous validation processes, ensuring consistent and precise analytical performance. The analytical features such as limit of detection (LOD), limit of quantification (LOC), linear working range and reproducibility were analysed under optimum condition. The linear working range was obtained by the calibration curve by measuring absorbance at different concentrations of ammonia. As seen in Fig. 10, the calibration curve of the sensor shows the coefficient of determination (*R*^2^) as 0.97 and the limit of detection of the developed sensor was calculated from the calibration curve by using formula as represented in eqn (5)5
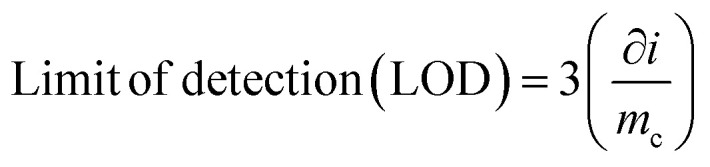
where ∂*i* refers to the standard deviation of blank sample and *m*_c_ refers the slope of linear range of calibration curve.

Similarly, the limit of quantification was calculated from the calibration curve by using the eqn (6)6
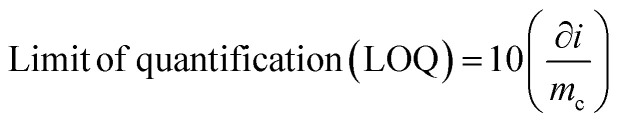


The linear working range of the sensor was 0 ppm to 100 ppm. The calculated values of limit of detection (LOD) and limit of quantification (LOQ) from calibration curve were obtained 8 ppm and 26 ppm respectively. The test for ammonia solution at 90 ppm were performed thrice to check the reproducibility and the percentage relative standard deviation (% RSD) of 2.78 is reported. The proposed sensor is designed to monitor ammonia in food packaging environments, where it is generated by protein degradation and spoilage. In such applications, ammonia concentrations typically increase to ppm levels or higher, making ultra-trace (ppb-level) detection less critical. Therefore, the achieved detection range is appropriate for practical spoilage monitoring.^67^

**Fig. 17:**
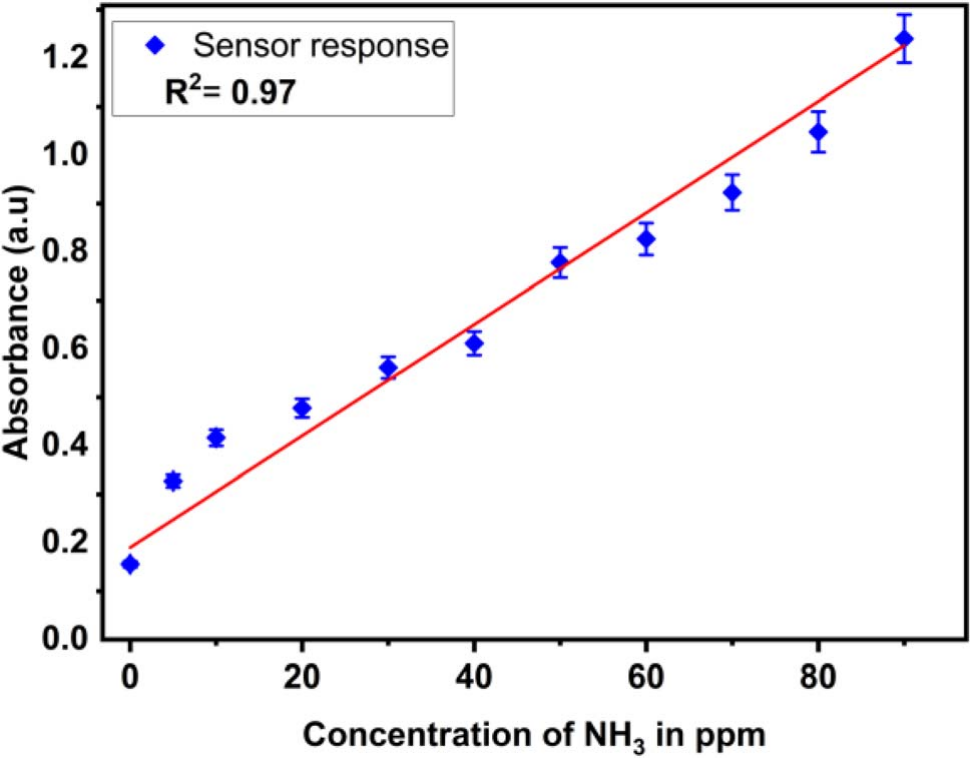
Calibration curve of ammonia sensor at different concentrations of 0 ppm to 90 ppm.

The concentration of ammonia is determined by Beer–Lambert law, which correlates the ammonia concentration with the absorbance of the light passing through the ammonia solution containing zinc oxide nanoparticle. As seen in the Fig. 17, the calibration curve obtained from the spectrophotometry method exhibits the linear working range of 0 ppm to 90 ppm with the coefficient of determination (*R*^2^) of 0.97.

### 3.11 Effect of biosynthesised ZnO nanoparticles on the optical properties and antimicrobial activity of bottlebrush extract-coated paper sensor

**Fig. 18:**
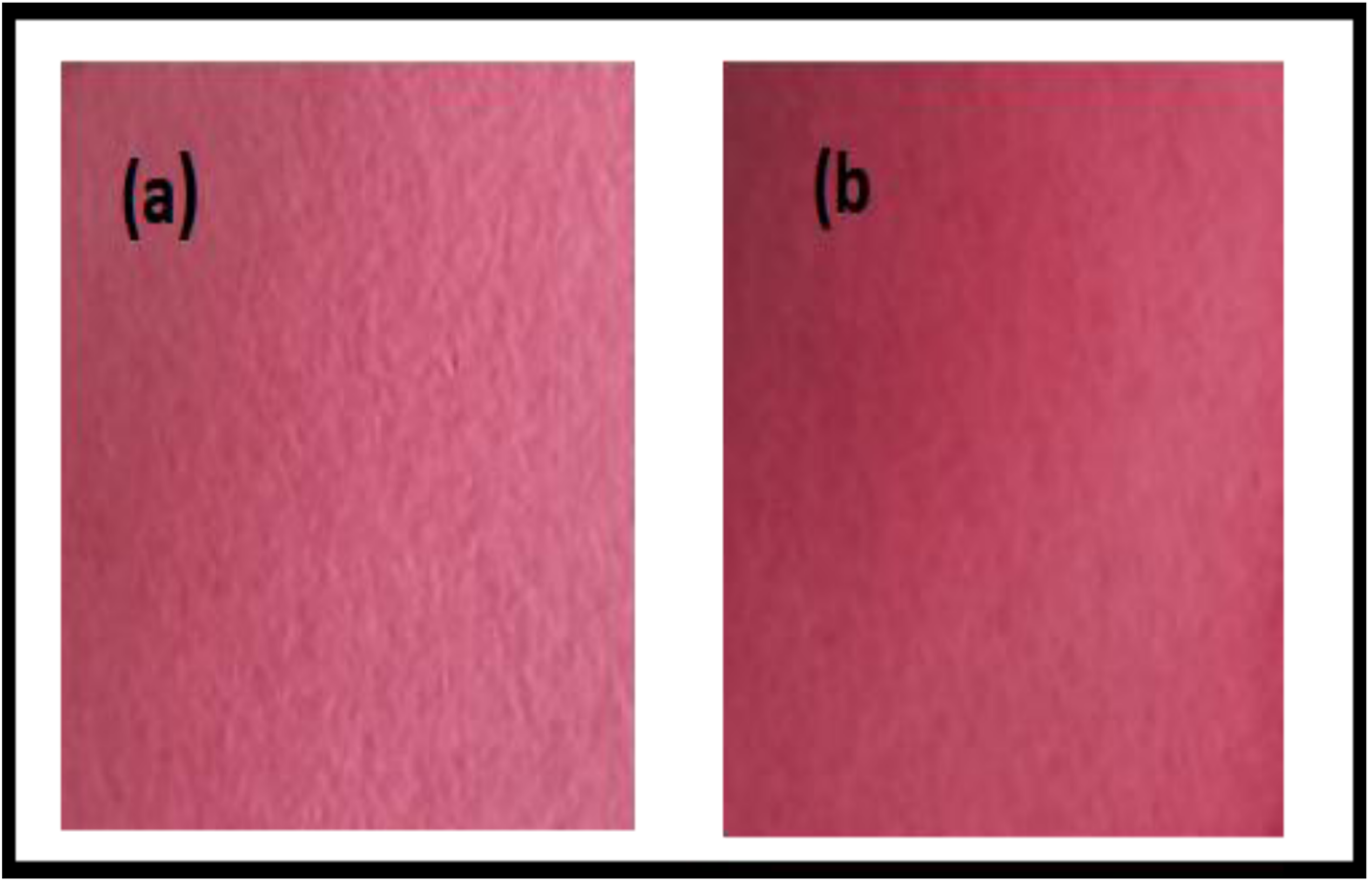
Visual appearance of the fabricated sensor (a) filter paper with bottlebrush extract, (b) filter paper with bottlebrush extract and ZnO NPs.

The visual appearance of the fabricated sensors is shown in Fig. 18. The sensor prepared with bottlebrush flower extract exhibited a light purple colour due to the presence of anthocyanins. Upon incorporation of biosynthesised ZnO nanoparticles (ZnO NPs), the colour changed to dark purple, indicating modification of the optical properties.

The corresponding CIELAB colour parameters (Table 4) show a decrease in lightness (L*), accompanied by an increase in a* (redness) and b* (yellowness) values after ZnO NP incorporation. These results indicate that the addition of ZnO NPs influences the optical characteristics of the anthocyanin-based sensor.

**Table 4:** Lab value of fabricated sensor with and without ZnO nanoparticles^a^

Sensor type	*L* value ± sd	*a*-value ± sd	*b*-value ± sd
Fabricated sensor without nanoparticles	51.75 ± 11.32	42.16 ± 4.74	6.233 ± 4.22
Fabricated sensor with ZnO nanoparticle	43.61 ± 11.82	44.73 ± 8.00	9.88 ± 3.38

a Sd = standard deviation.

ZnO nanoparticles exhibit antimicrobial activity by inhibiting bacterial growth on the filter paper. In addition, anthocyanins possess inherent antimicrobial properties. The combined effect enhances resistance against spoilage microorganisms, as shown in Fig. 15.

#### 3.11.1 Chicken freshness color response test

Chicken is susceptible to microbial degradation, leading to the formation of volatile basic nitrogen compounds, including ammonia, along with lipid oxidation products during storage.

**Fig. 19:**
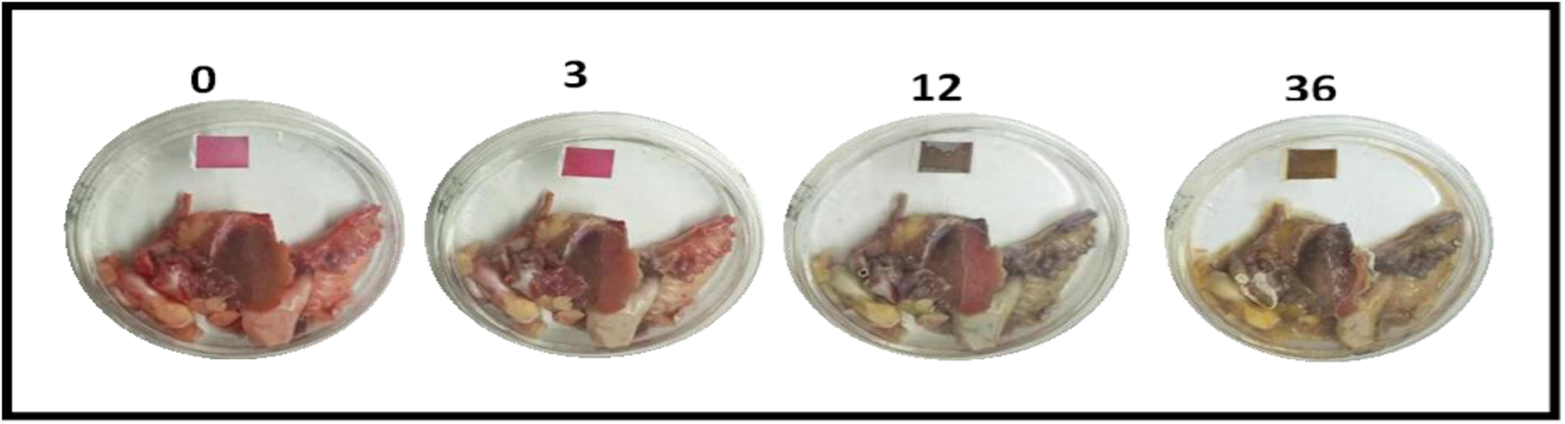
Degradation of chicken at different time interval.

**Fig. 20:**
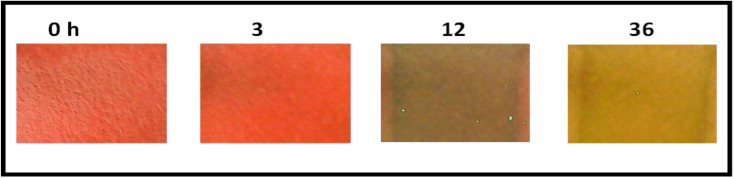
Degradation of chicken at different time interval images taken by digital microscope.

The CIELAB colour parameters (Fig. 19) show progressive changes with storage time, indicating deterioration of the chicken samples. The corresponding colourimetric response of the fabricated ZnO-based sensor, obtained from digital microscope images (Fig. 20) and analysed using image processing, is presented in Table 5.

**Table 5:** Lab value of chicken spoilage at different time interval^a^

Time (h)	*L* value ± sd	*a*-value ± sd	*b*-value ± sd
0	57.71 ± 0.002	47.175 ± 0.007	40.516 ± 0.13
3	55.73 ± 0.028	53.894 ± 0.004	49.75 ± 0.03
12	48.65 ± 0.087	10.14 ± 0.13	32.56 ± 0.17
36	59.23 ± 0.036	10.36 ± 0.044	58.88 ± 0.044

a Sd = standard deviation.

The visual changes in chicken samples and the corresponding response of the fabricated sensor over time (0–36 h) provide a clear indication of freshness and suitability for consumption. At 0 h, the chicken exhibits a characteristic bright red appearance, and the sensor shows a pink colour, indicating a fresh sample that is suitable for consumption. At 3 h, only minor surface changes are observed in the chicken, and the sensor colour remains pink, suggesting that the sample is still acceptable for consumption, although early changes may have initiated.

At 12 h, noticeable deterioration in the chicken is evident, with a darker and duller appearance. The sensor colour shifts to a greyish tone, indicating the onset of spoilage. This transition is associated with microbial activity and the production of volatile basic nitrogen compounds, such as ammonia, which increase the pH and interact with the anthocyanin-based sensing matrix. At this stage, the sample is considered unsuitable for consumption.

At 36 h, the chicken shows clear signs of advanced spoilage, including yellowish discolouration and structural degradation. The sensor exhibits a brown colour, reflecting a high level of spoilage due to the accumulation of degradation products and continued microbial activity. Under these conditions, the sample is clearly unsafe and should be discarded.

Overall, the sensor demonstrates a distinct and time-dependent colour transition that correlates well with the progression from fresh to spoiled states, enabling rapid visual assessment of chicken quality and providing a practical indication of whether the sample is suitable for consumption or should be discarded. The CIELAB colour parameters show clear time-dependent changes during storage, reflecting progressive spoilage of the chicken samples.

At 0 h, the sample exhibited high L*, a*, and moderate b* values, indicating fresh meat characteristics. At 3 h, a slight decrease in L* was observed, while a* and b* values increased, suggesting initial changes in the sample. At 12 h, both L* and a* values decreased significantly, indicating darkening and loss of red colour associated with spoilage progression, along with a decrease in b*. At 36 h, L* increased, while a* remained low and b* increased markedly, indicating pronounced yellowing at advanced stages of spoilage. These variations in L*, a*, and b* values indicate progressive deterioration of the chicken samples with storage time.

## 4. Conclusion

In conclusion, the bio-inspired synthesis of zinc oxide nanoparticles using bottole brush flower offers a promising approach to enhance the development of advanced sensors for ammonia detection and food quality monitoring. UV-visible spectroscopy confirmed the biosynthesized ZnO nanoparticles peak at 383 nm. FT-IR spectra reveals that the related functional groups are present. Biosynthesized ZnO nanoparticles are used for ammonia sensing and the antioxidant activity of ZnO nanoparticles is also reported. The biosynthesized zinc oxide nanoparticles show high antioxidant activity even at lower concentrations. The ZnO NPs shows a free radical scavenging capacity of up to 71%, for biosynthesized zinc oxide nanoparticle whereas bottole flower antioxidant scavenging capacity was found upto 63% which leads to a better antioxidant activity. The fabricated ZnO NPs demonstrate high sensitivity and selectivity towards ammonia, making them highly suitable for industrial applications. Furthermore, their integration in food quality monitoring systems shows potential for ensuring food safety by detecting spoilage at early stages. This innovative synthesis method not only highlights the advantages of bio-inspired materials in nanotechnology but also paves the way for sustainable, cost-effective, and efficient solutions in sensing applications. Further research is needed to optimize the sensor devices and explore other potential applications of bio-derived nanomaterials in environmental monitoring and beyond.

## Conflicts of interest

There are no conflicts to declare.

## Data Availability

The data that support the findings of this study are reported in this manuscript.
